# Evaluation of 3-tier and 5-tier FHR pattern classifications using umbilical blood pH and base excess at delivery

**DOI:** 10.1371/journal.pone.0228630

**Published:** 2020-02-06

**Authors:** Hitomi Kikuchi, Shunichi Noda, Shinji Katsuragi, Tomoaki Ikeda, Hiroyuki Horio

**Affiliations:** 1 Department of Medical Engineering, Aino University, Ibaraki, Osaka, Japan; 2 Noda Clinic, Miyakonojo-shi, Miyazaki, Japan; 3 Department of Obstetrics and Gynecology, Sakakibara Heart Institute, Fuchu-shi, Tokyo, Japan; 4 Department of Obstetrics and Gynecology, Mie University Graduate School of Medicine, Tsu, Mie, Japan; 5 Graduate School of Applied Informatics, University of Hyogo, Kobe, Hyogo, Japan; Vanderbilt University Medical Center, UNITED STATES

## Abstract

**Objective:**

The relevance between time-series fetal heart rate (FHR) pattern changes during labor and outcomes such as arterial blood gas data at delivery has not been studied. Using 3-tier and 5-tier classification systems, we studied the relationship between time-series FHR pattern changes before delivery and umbilical artery blood gas data at delivery.

**Methods:**

The subjects were 1,909 low-risk women with vaginal delivery (age: 29.1 ± 4.4 years, parity: 1.7 ± 0.8). FHR patterns were classified by a skilled obstetrician based on each 10 min-segment of the last 60 min before delivery from continuous CTG records in an obstetric clinic.

**Results:**

The relationship between each 10 min-segment FHR pattern classification from 60 minutes before delivery and umbilical artery blood pH and base excess (BE) values at delivery changed with time. In the 3-tier classification, mean pH of Category I group in each 10 min-segment was significantly higher than that of Category II group. For Category I groups in each 10-minute segment, its number decreased and its average pH increased as the delivery time approached. In the 5-tier classification, there was the same tendency. About each level group in 10 min-segment, the higher the level, the lower the blood gas values, and mean pH of higher level groups decreased as the delivery time approached.

**Conclusions:**

The relationship between classifications and outcomes was clear at any time from 60 min before delivery in 3- and 5-tier classifications, and the 5-tier classification was more relevant.

## Introduction

In cardiotocography (CTG), fetal conditions are monitored by continuous measurement of fetal heart rate (FHR) and uterine contractions. This monitoring provides nearly real-time fetal well-being from heart rate changes and is used in 89% of delivery cases in the United States (2004) [1], 91% in Canada (2009) [2], approximately 60% in the United Kingdom (UK) as National Institute for Health and Care Excellence (NICE) guidelines for fetal monitoring in the NHS detail explicit criteria for this monitoring (2018) [3]. In Japan, it is estimated to be almost 100%.

FHR waveforms are classified by four indicators: baseline, baseline variability, accelerations, and decelerations. Intrapartum guidelines using 3-tier and 5-tier classification systems to estimate fetal hypoxia and acidosis level have been proposed in many countries [4–8]. At present, the 3-tier classification based on FHR patterns is used in the United States, Canada, and UK. The classification in the United States is defined as Category I, II, III, and in Canada, as Normal, Atypical, and Abnormal [7]. In the UK, there is a 3-tier description classified as Reassuring, Non-reassuring, and Abnormal. The management categories for labor based on the 3-tier FHR pattern interpretations are defined as: Normal, Suspicious, Pathological, and Need for urgent interventions [4, 5]. A 5-tier classification was proposed in Japan [8] because the 3-tier classification was considered to be simple, with Category II covering too wide a range.

Three or five tier management guidelines based on the classification of FHR patterns are used in each country. At present, although there are many studies around the effectiveness of FHR pattern classification and outcomes [9–14], the effectiveness of continuous cardiotocography has not been sufficiently shown in comparison with intermittent auscultation [15, 16]. In these studies, the relevance between time-series changes in FHR pattern classification during labor and outcomes such as arterial blood gas data at delivery has not been studied. Therefore, we tried to analyze the relationship using the U.S. 3-tier and Japanese 5-tier guidelines between each 10 min-segment FHR pattern change from 60 minutes before delivery and umbilical artery blood gas data at delivery.

## Materials and methods

The subjects in this study were 1,909 low-risk women with vaginal delivery (age: 29.1 ± 4.4 years, parity: 1.7 ± 0.8) from 2003 to 2006 at an obstetric clinic (Table 1). The data used in our study was fully anonymized before offered. A skilled obstetrician who was blinded to outcome retrospectively classified each 10 min-segment of the last 60 min CTG data before delivery using the framework of Parer et al [17]. The outcomes were umbilical artery blood pH and base excess (BE) at delivery. The classified data were applied to the 3-tier classification used in the United States (Table 2) and the 5-tier classification used in Japan (Table 3). We analyzed the relationship between time-series changes of FHR patterns classification before delivery and outcomes at delivery. Another analysis was a grouping of outcomes, which was as follows: pH <7.0, 7.0≤ pH <7.1, 7.1≤ pH <7.2, 7.2≤ pH <7.3, 7.3≤ pH <7.4, pH ≥7.4, and BE <-12, BE ≥-12.

**Table 1:** Patient characteristics.

Item	Mean ± SD (n = 1909)
Gestational age	39 w 5 d ± 10 d
Duration of labor	7 h 59 m ± 6 h 4 m
Instrumental delivery (%)	25
Oxytocin administration (%)	5.6
Maternal age (years)	28.9 ± 4.4
Parity (time)	1.7 ± 0.8
Fetal state	
Birth Weight (g)	3210 ± 85
Gender (%)	Male: 52, Female: 48
Apgar score 1 minute	9.24 ± 0.56
Apgar score 5 minute	9.57 ± 0.56

**Table 2:** Summary of 3-tier FHR pattern classification.

Category	FHR tracing
**Category I**	Baseline rate: 110–160 beats per minute
	Baseline variability: moderate
	Late or variable decelerations: absent
	Early decelerations: present or absent
	Accelerations: present or absent
**Category II**	Includes all tracings not categorized as Category I or III
**Category III**	Absent baseline FHR variability and any of the following • Recurrent late decelerations • Recurrent variable decelerations • Bradycardia
	Sinusoidal pattern

**Table 3:** Summary of 5-tier FHR pattern classification.

Baseline FHR		None	Early	Variable		Late		Prolonged	
				Mild	Severe	Mild	Severe	Mild	Severe
**Moderate variability (amplitude 6–25 bpm)**	110–160 bpm	1	2	2	3	3	3	3	4
	>160 bpm	2	2	3	3	3	4	3	4
	80–110 bpm	3	3	3	4	4	4	4	4
	<80 bpm	4	4		4	4	4		
**Minimal variability (amplitude 3–5 bpm)**	110–160 bpm	3	3	3	4	3	4	4	
	>160 bpm	3	3	4	4	4	5	4	
	80–110 bpm	4	4	4	5	5	5	5	5
	<80 bpm	5	5		5	5	5		
**Undetectable variability (amplitude ≤2 bpm)**		4	5	5	5	5	5	5	5
**Marked variability (amplitude ≥26 bpm)**				3	3	3	4	3	4
**Sinusoidal FHR pattern**		4	4	4	4	5	5	5	5

1: Level 1, 2: Level 2, 3: Level 3, 4: Level 4, 5: Level 5.

Statistical analysis was performed by Friedman, Tukey’s HSD, Wilcoxon signed-rank and Kruskal-Wallis test for these four respective indicators, using JMP (ver. 10, SAS) with a significance level of 0.05. The study was approved by the research ethics committee of the Graduate School of Applied Informatics, University of Hyogo.

## Results

The frequency of the 3- and 5-tier classifications in each 10 min-segment of the last 60 min before delivery are shown in Table 4. In the 3-tier classification, Category I decreased as labor proceeded, while Category II significantly increased. The incidence of Category III, which may indicate fetal acidosis, was only 0.1% (n = 2) from 50 to 30 min before delivery. In the 5-tier classification, the incidences of Level 1 and 2, categorized as normal FHR pattern, significantly decreased as labor proceeded, whereas the incidences of Level 3 to 5, which may indicate fetal acidosis, significantly increased.

**Table 4:** The frequency of 3-tier (category) and 5-tier (level) classifications in each 10 min-segment of the last 60 min before delivery.

Time before delivery		60 min	50 min	40 min	30 min	20 min	10 min
**3-tier**	Category I	55.5%	50.6%	47.2%	36.7%	23.5%	1.9%
	Category II	44.5%	49.3%	52.6%	63.2%	76.5%	98.1%
	Category III	0.0%	0.1%	0.1%	0.1%	0.0%	0.0%
**5-tier**	Level 1	54.0%	49.3%	46.2%	35.9%	22.9%	1.9%
	Level 2	34.5%	38.0%	39.0%	46.0%	51.2%	35.7%
	Level 3	10.7%	11.6%	13.8%	16.3%	22.5%	44.5%
	Level 4	0.7%	1.0%	1.0%	1.7%	3.2%	17.5%
	Level 5	0.1%	0.0%	0.0%	0.1%	0.2%	0.4%

**Fig 1:**
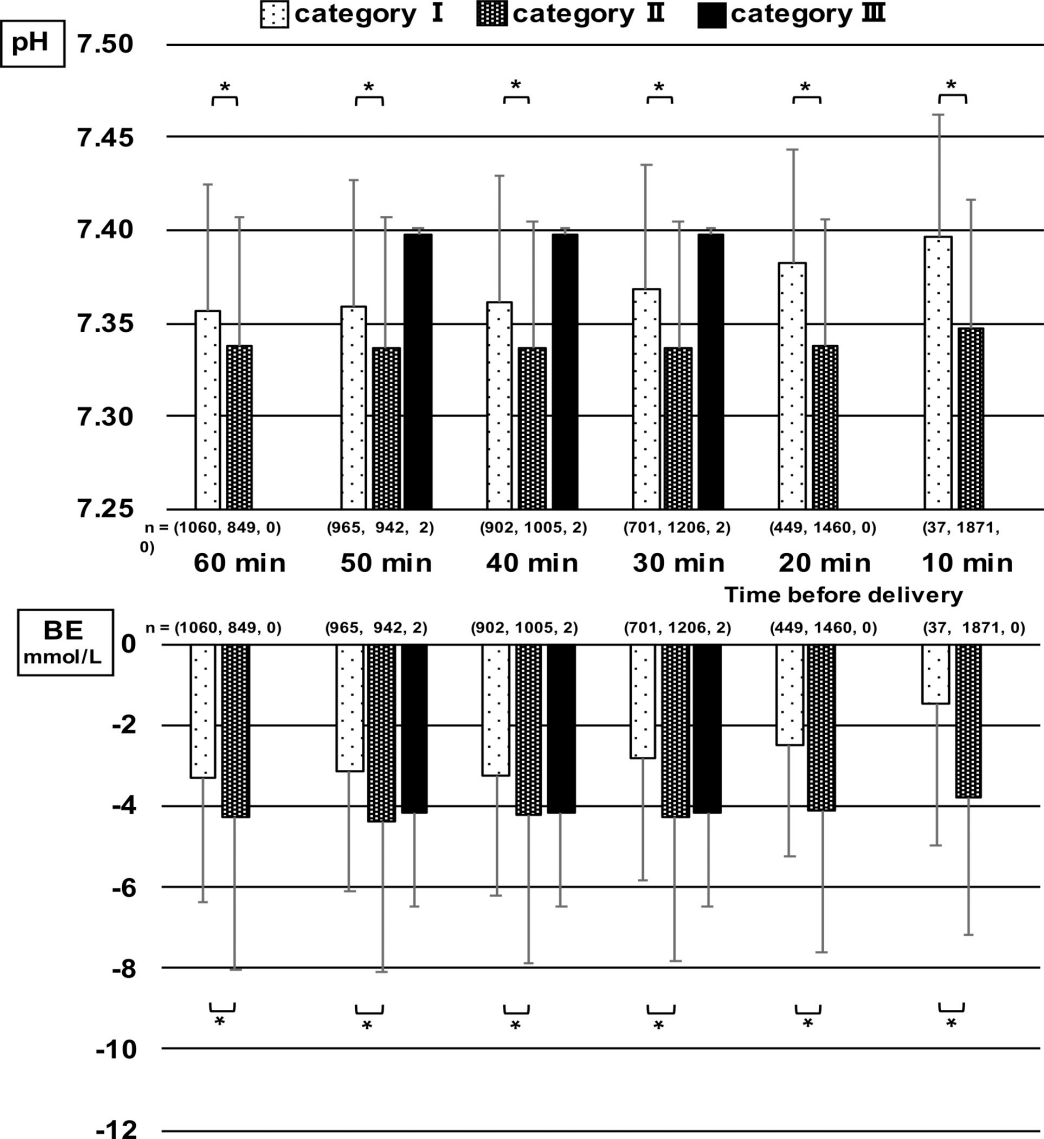
Relationships of umbilical artery blood pH and BE at delivery with 3-tier classification in each 10 min-segment of the last 60 min before delivery (mean + standard deviation). There were significant differences between category I and category II.

**Fig 2:**
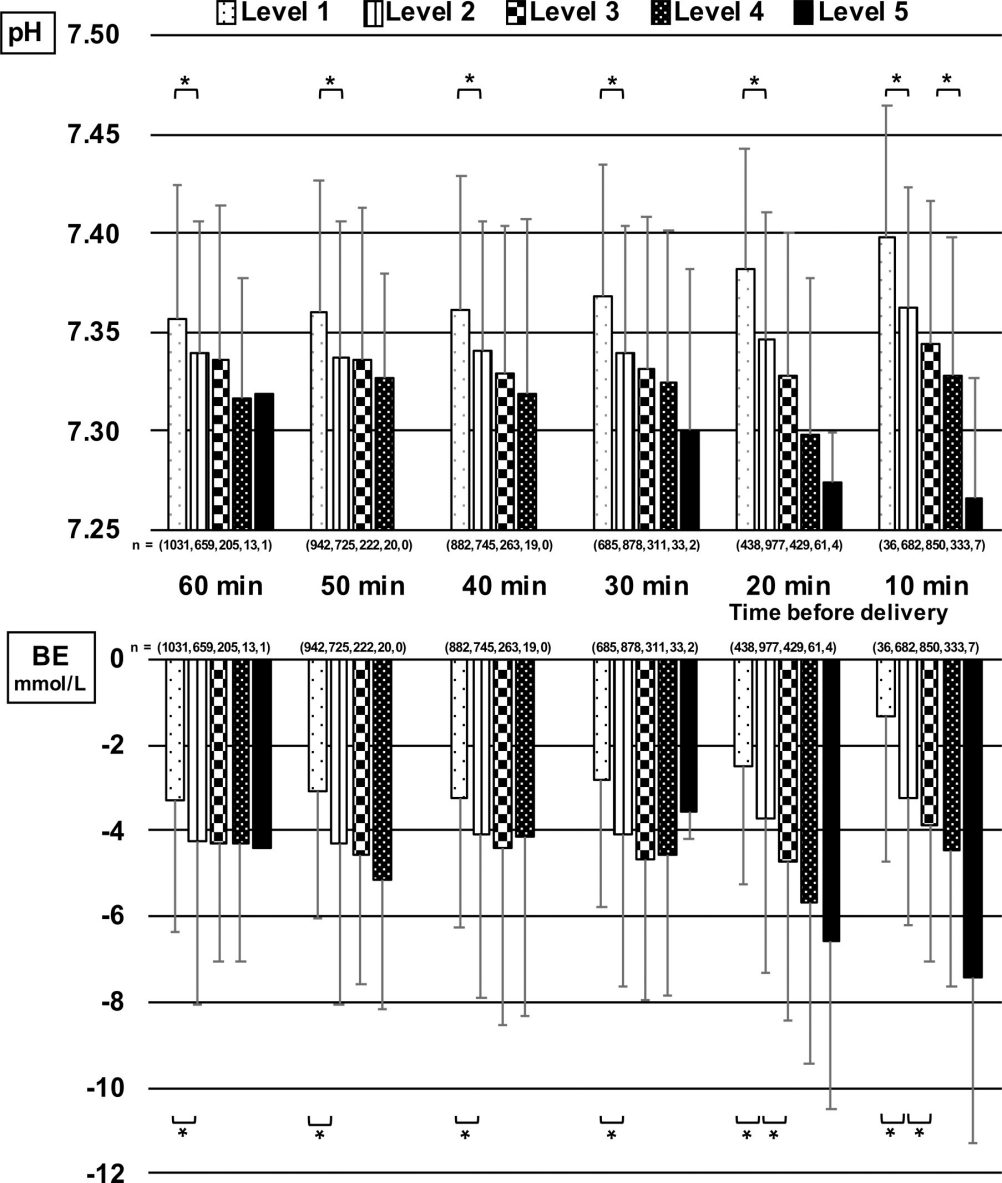
Relationships of umbilical artery blood pH and BE at delivery with 5-tier classification in each 10 min-segment of the last 60 min before delivery (mean + standard deviation). There were significant differences between Level 1 and Level 2.

The relationship between the 3-tier classification in each 10 min-segment of the last 60 min before delivery and umbilical artery blood pH and BE at delivery are shown in Fig 1 and those for the 5-tier classification are shown in Fig 2. The horizontal axis of each figure shows 3- and 5-tier classifications in each 10 min-segment of the last 60 min before delivery. The vertical axis shows the mean and standard deviations of related outcome data. In the 3-tier classification, the relationship of Category I with umbilical artery blood pH at delivery was significantly higher than that of Category II, and the relationship of Category I with BE at delivery was significantly higher than that of Category II. There was no significant difference between Categories II and III because of the small number of cases in Category III (n = 2) from 50 to 30 min before delivery. In the 5-tier classification, pH showed a tendency to decrease as the level became higher at any 10 min-segment from 60 min before delivery, which showed the possibility of fetal acidosis. Significant differences in pH were found between Level 1 and 2 from 60 to 10 min before delivery. BE began to decrease at 20 min before delivery and there were significant differences in BE between Level 1 and 2, and Level 2 and 3.

**Fig 3:**
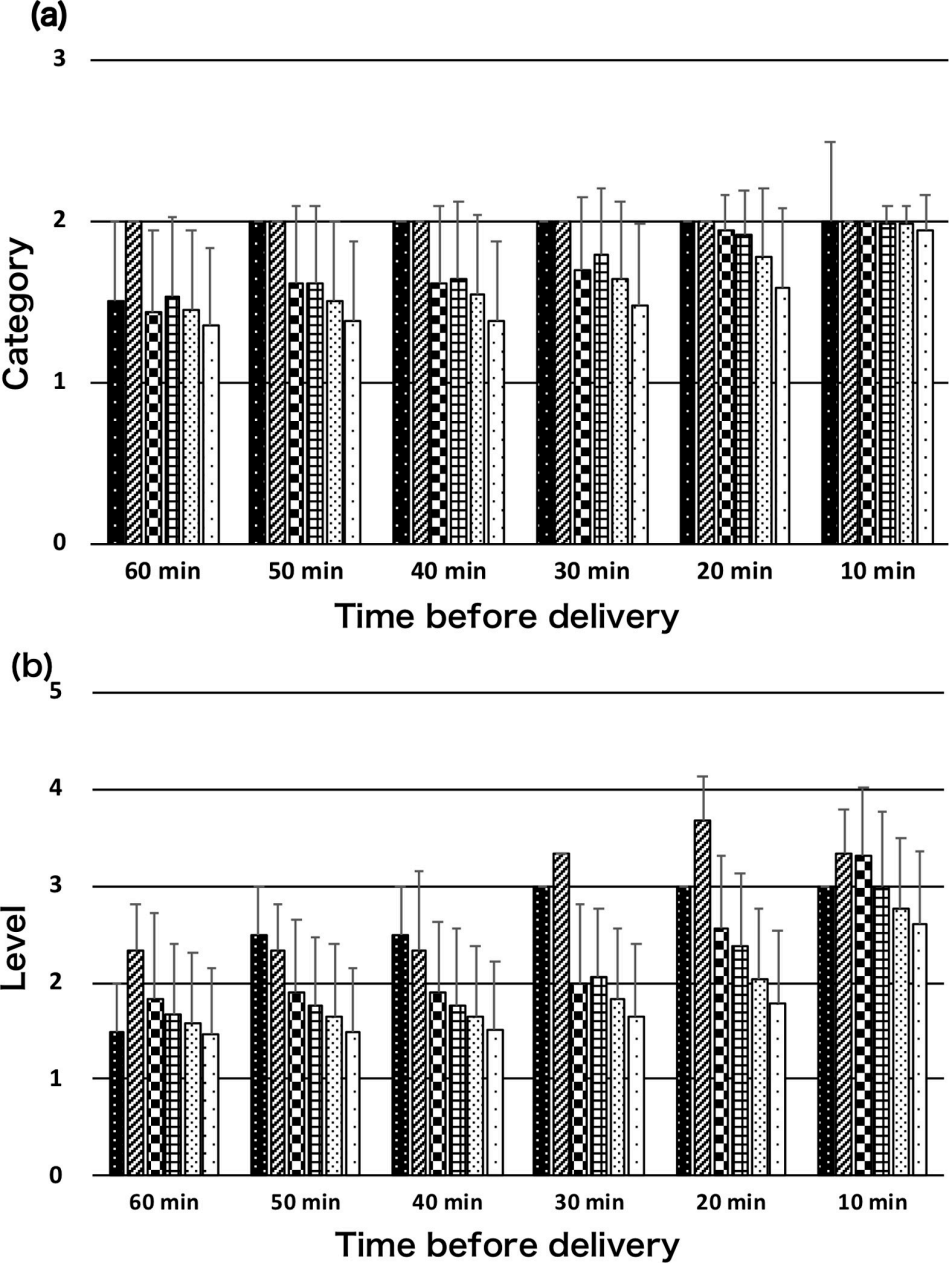
Relationships of mean 3-tier (a) and mean 5-tier (b) classification values with pH groups at delivery in each 10 min-segment of the last 60 min before delivery.

Of the 1,909 subjects, 2 (0.1%) had pH <7.0, and 3 (0.2%) had 7.0≤ pH <7.1, and 36 (1.9%) had 7.1≤ pH <7.2, and 372 (19.5%) had 7.2≤ pH <7.3, 1080 (56.6%) had 7.3≤ pH <7.4, and 416 (21.8%) had pH ≥7.4. Regarding BE (mmol/L), BE <-12 was 21 (1.1%), and BE ≥-12 was 1888 (98.9%). Fig 3 shows the relationships of these groups with mean 3-tier (a) and mean 5-tier (b) classification values with pH groups at delivery in each 10 min-segment of the last 60 min before delivery. There was a significant difference among mean classification values within the same time in both 3-tier and 5-tier groups. However, in part, there was no difference among them. Table 5 shows mean 3-tier and mean 5-tier classification values with BE <-12 mmol/L and BE ≥-12 mmol/L groups at delivery in each 10 min-segment of the last 60 min before delivery. The mean 3-tier classification value of BE <-12 mmol/L group significantly tended to be higher except 30 and 10 min before delivery. The mean 5-tier classification value of BE <-12 mmol/L group always showed significantly higher than BE ≥-12 mmol/L group. The mean value of both groups tended to higher with time to delivery.

**Table 5:** Relationships of mean 3-tier and mean 5-tier classification values with BE groups at delivery in each 10 min-segment of the last 60 min before delivery.

	Level	BE<-12mmol/L	BE≧-12mmol/L	Wilcoxon p-value
	n	21	1888	
**3-tier**	60min	1.71	1.44	0.0125
	50min	1.86	1.49	0.0009
	40min	1.81	1.53	0.0095
	30min	1.81	1.63	0.0931
	20min	1.95	1.76	0.0416
	10min	2.00	1.98	0.5274
**5-tier**	60min	2.05	1.58	0.0039
	50min	2.19	1.64	0.0003
	40min	2.14	1.69	0.0070
	30min	2.33	1.84	0.0047
	20min	2.71	2.06	0.0004
	10min	3.20	2.78	0.0222

## Discussion

The interpretation of FHR pattern in this study was performed by a single examiner with more than 20 years of clinical experience who was blinded to outcome. There are two factors contribute to the agreement level of FHR pattern interpretation by examiner: inter-observer agreement between multiple examiners, and intra-observer agreement with repeated examinations by a single examiner. In addition, in the case of multiple examiners, the agreement level of repeat examinations is also involved. As for the agreement level in FHR pattern interpretations, the level of repeat examinations by a single examiner was higher than that among multiple examiners [18], and the agreement level among multiple examiners was widely dispersed [19]. Therefore, the interpretation of FHR patterns in this study was performed by a single examiner.

Guideline for FHR pattern interpretation in Japan uses a 5-tier classification [8], whereas a 3-tier classification is used in many other countries [4–7, 20]. From the results, the tendency of the frequency in both 3- and 5-tire classifications in each 10 min-segment of the last 60 min before delivery showed that higher-level classifications increased and lower-level classifications decreased over time. With regard to the relationship between classification categories at each 10 min-segment from 60 min before delivery and umbilical artery blood gas data at delivery, one was the relationship between the 3- and 5-tier classification pattern and the outcome at each segment, and the other was the analysis of mean classification values among the groups divided by outcome level. These were complementary to each other. The results showed that poor FHR pattern group had a poor outcome, and on the contrary, poor outcome group had a poor FHR pattern. This tendency continued from 60min before delivery. The relation between the outcome pH groups and the average classification values with each group showed that the groups with lower pH values in both 3-tier and 5-tier had significantly higher classification values over time, and the mean values also tended to increase over time. The BE group divided into two groups had the same tendency and this is more clear in the 5-tier classification.

No literature has been found about the relationship between changes in FHR pattern over time and outcomes. A similar study [21] of the relationship between FHR classification and pH at delivery showed that the sensitivity to fetal acidosis in the 5-tier classification was higher than that in the 3-tier classification. This tendency was confirmed in the current study from 60 min before delivery, with BE showing a similar tendency. These results suggest that the 5-tier classification at any time from 60 min before delivery have a closer relationship with umbilical artery blood gas data at delivery, compared to the 3-tier classification. As in Coletta et al [21, 22], comparing fetus groups with pH <7.0 and pH >7.2, the result of 3-tier and 5-tier classifications showed that the group with higher pH gathered in the lower levels, and those with lower pH gathered in the higher categories. However, this is the result of cumulative time course of FHR classification, because the concept of time course essential for finding the tendency is sparse. In our analysis, we could clarify the relationship between changes in 3- or 5-tier classification along the time-series analysis from 60 minutes before delivery and the outcomes.

The purpose of analyzing the characteristics of FHR waveform pattern is to find evidence of prognostic signs in the process from hypoxemia to metabolic acidosis occurred during labor. The 3-tier or 5-tier classification for continuous FHR monitoring is considered an integrated method for that purpose in which the presence of acceleration and moderate FHR variability can predict the absence of metabolic acidosis [23]. However, there is little concept of time course in the current classifications. In this study, we have analyzed under the concept of time-series FHR classification. The results showed that poor FHR classification group had a poor outcome, and on the contrary, poor outcome group had a poor FHR pattern from 1 hour before delivery. The 5-tier classification became clearer than the 3-tier classification using time-series analysis. Although this result is from normal cases, we expect to find characteristic signs that lead to early detection of fetal acidosis from the time-series changes in FHR data.

## Conclusion

The result from the analysis between time-series changes of FHR pattern classification before delivery and outcomes at delivery showed that the relationship between classifications and outcomes was clear at any time from 60 min before delivery in 3- and 5-tier classifications, and that the 5-tier classification was more relevant.

## References

[1] Chen HY, Chauhan SP, Ananth CV, Vintzileos AM, Abuhamad AZ. Electronic fetal heart rate monitoring and its relationship to neonatal and infant mortality in the United States. Am J Obstet Gynecol. 2011;204: 491.e1-10. 10.1016/j.ajog.2011.04.024 21752753

[2] Public Health Agency of Canada. What Mothers Say: The Canadian Maternity Experiences Survey. Ottawa, 2009. Available at: http://www.publichealth.gc.ca/mes. Accessed October 30, 2017.

[3] Brocklehurst P, Field D, Greene K, Juszczak E, Kenyon S, Linsell L, et al Computerised interpretation of the fetal heart rate during labour: a randomised controlled trial (INFANT). Health Technol Assess. 2018;22(9). 10.3310/hta22090 29437032

[4] National Collaborating Centre for Women's and Children's Health (UK). Intrapartum Care: Care of Healthy Women and Their Babies During Childbirth. NICE Clinical Guidelines, No. 190. London: National Institute for Health and Care Excellence (UK); 2014. 25950072

[5] NICE: Intrapartum care for healthy women and babies (CG190). Feb 2017. Available from https://www.nice.org.uk/guidance/cg190 Cited 08 June 2019.

[6] American College of Obstetricians and Gynecologists. ACOG Practice Bulletin No.116: Management of Intrapartum Fetal Heart Rate Tracings. Obstet Gynecol. 2010;116: 1232–1240. 10.1097/AOG.0b013e3182004fa9 20966730

[7] Liston R, Sawchuk D, Young D. Society of Obstetrics and Gynecologists of Canada, British Columbia Perinatal Health Program. Fetal health surveillance: Antepartum and intrapartum consensus guideline. J Obstet Gynaecol Can. 2007;29: S3–S56. 17845745

[8] Okai T, Ikeda T, Kawarabayashi T, Kozuma S, Sugawara J, Chisaka H, et al Intrapartum management guidelines based on fetal heart rate pattern classification. J Obstet Gynaecol Res. 2010;36: 925–928. 10.1111/j.1447-0756.2010.01342.x 21058434

[9] Dellinger EH, Boehm FH, Crane MM. Electronic fetal heart rate monitoring: Early neonatal outcomes associated with normal rate, fetal stress, and fetal distress. Am J Obstet Gynecol. 2000;182: 214–220. 10.1016/s0002-9378(00)70515-1 10649181

[10] Parer JT, King T, Flanders S, Fox M, Kilpatrick SJ. Fetal acidemia and electronic fetal heart rate patterns: is there evidence of an association? J Matern Fetal Neonatal Med. 2006; 19: 289–294. 10.1080/14767050500526172 16753769

[11] Sameshima H, Ikenoue T, Ikeda T, Kamitomo M, Ibara S. Unselected low-risk pregnancies and the effect of continuous intrapartum fetal heart rate monitoring on umbilical blood gases and cerebral palsy. Am J Obstet Gynecol. 2004;190: 118–123. 10.1016/j.ajog.2003.07.014 14749646

[12] Sameshima H, Ikenoue T. Predictive value of late decelerations for fetal acidemia in unselective low-risk pregnancies. Am J Perinatol. 2005;22: 19–23. 10.1055/s-2004-837272 15668840

[13] Sadaka A, Furuhashi M, Minami H, Miyazaki K, Yoshida K, Ishikawa K. Observation on validity of the five-tier system for fetal heart rate pattern interpretation proposed by Japan Society of Obstetricians and Gynecologists. J Maternal-Fetal Neonatal Med. 2011;24: 1465–1469. 10.3109/14767058.2011.621999 21923306

[14] Cahill AG, Tuuli MG, Stout MJ, López JD, Macones GA. A prospective cohort study of fetal heart rate monitoring: deceleration area is predictive of fetal acidemia. Am J Obstet Gynecol. 2018;218(5): 523.e1-523.e12. 10.1016/j.ajog.2018.01.026 PMC5916338 29408586

[15] Alfirevic Z, Devane D, Gyte GM, Cuthbert A. Continuous cardiotocography (CTG) as a form of electronic fetal monitoring (EFM) for fetal assessment during labour. Cochrane Database Syst Rev. 2017 2 3;2: CD006066 10.1002/14651858.CD006066.pub3 16856111

[16] Devane D, Lalor JG, Daly S, McGuire W, Cuthbert A, Smith V. Cardiotocography versus intermittent auscultation of fetal heart on admission to labour ward for assessment of fetal wellbeing. Cochrane Database Syst Rev. 2017 1 26; 1: CD005122 Review. 10.1002/14651858.CD005122.pub5 28125772 PMC6464914

[17] Parer JT, Ikeda T. A framework for standardized management of intrapartum fetal heart rate patterns. Am J Obstet Gynecol. 2007;197: 26.e1-e6. 10.1016/j.ajog.2007.03.037 17618744

[18] Paneth N, Bommarito M, Stricker J. Electric fetal monitoring and later outcome. Clin Invest Med. 1993;16: 159–65. 8513616

[19] Blackwell SC, Grobman WA, Antoniewicz L, Hutchinson M, Gyamfi BC. Interobserver and intraobserver reliability of the NICHD 3-Tier Fetal Heart Rate Interpretation System. Am J Obstet Gynecol. 2011;205: 378.e1-5. 10.1016/j.ajog.2011.06.086 21864826

[20] Ayres-de-Campos D, Spong CY, Chandraharan E. FIGO consensus guidelines on intrapartum fetal monitoring: Cardiotocography. Int J Gynaecol Obstet. 2015; 131: 13–24. 10.1016/j.ijgo.2015.06.020 26433401

[21] Coletta J, Murphy E, Rubeo Z, Gyamfi-Bannerman C. The 5-tier system of assessing fetal heart rate tracings is superior to the 3-tier system in identifying fetal academia. Am J Obstet Gynecol. 2012;206: 226.e1-e5. 10.1016/j.ajog.2011.12.014 22244473

[22] Miller DA, Miller LA. Three-tier versus five-tier fetal heart rate classification systems. Am J Obstet Gynecol. 2012;207(6): e8–9. 10.1016/j.ajog.2012.07.014 22857916

[23] Macones GA, Hankins GD, Spong CY, Hauth J, Moore T. The 2008 National Institute of Child Health and Human Development workshop report on electronic fetal monitoring: update on definitions, interpretation, and research guidelines. Obstet Gynecol. 2008;112(3): 661–6. 10.1097/AOG.0b013e3181841395 18757666

